# Epigenetic regulation of cholesterol homeostasis

**DOI:** 10.3389/fgene.2014.00311

**Published:** 2014-09-24

**Authors:** Steve Meaney

**Affiliations:** ^1^School of Biological Sciences, College of Sciences and Health, Dublin Institute of TechnologyDublin, Ireland; ^2^Environmental Sustainability and Health Institute, Dublin Institute of TechnologyDublin, Ireland

**Keywords:** cholesterol, oxysterol, HMGCR, PCSK9, LDLR, APOE, APOJ, CYP46A1

## Abstract

Although best known as a risk factor for cardiovascular disease, cholesterol is a vital component of all mammalian cells. In addition to key structural roles, cholesterol is a vital biochemical precursor for numerous biologically important compounds including oxysterols and bile acids, as well as acting as an activator of critical morphogenic systems (e.g., the Hedgehog system). A variety of sophisticated regulatory mechanisms interact to coordinate the overall level of cholesterol in cells, tissues and the entire organism. Accumulating evidence indicates that in additional to the more “traditional” regulatory schemes, cholesterol homeostasis is also under the control of epigenetic mechanisms such as histone acetylation and DNA methylation. The available evidence supporting a role for these mechanisms in the control of cholesterol synthesis, elimination, transport and storage are the focus of this review.

## INTRODUCTION

Cholesterol (cholest-5-ene-3β-ol) is an ubiquitous 27-carbon steroid and a vital component of cellular membranes in vertebrates (Vance and Vance, 2002). It is essential for life, and defects in the ability to synthesize it leads to severe clinical conditions such as the Smith–Lemli–Opitz syndrome and desmosterolosis (Porter, 2003). In man the total pool of cholesterol is 120–130 g and as it is a neutral, hydrophobic lipid the vast majority is found as the free sterol in the membrane (Cook, 1958). The distribution of cholesterol across organs, cells, and subcellular membranes is not even, with some organs (e.g., the brain) and cell types (e.g., oligodendroglia) highly enriched in cholesterol. Within the cell, the low cholesterol content in intracellular membranes facilitates cholesterol sensing and homeostatic feedback (Steck and Lange, 2010; Jeon and Osborne, 2012). In addition to this essential structural role cholesterol is also a precursor for numerous biomolecules of physiological importance including bile acids, steroid hormones, and oxysterols^1^.

All nucleated cells can synthesize cholesterol. As a consequence, under normal circumstances there is no requirement to ingest cholesterol. However, a typical western diet contains approximately 300–500 mg of cholesterol per day and so cholesterol originates from either *de novo* synthesis or dietary intake (Wang, 2007). This cholesterol is absorbed in the small intestine and is distributed throughout the body via the action of lipoproteins to most, but not all, tissues. The brain is a notable exception – cholesterol is unable to pass the blood–brain barrier and cholesterol in the brain is due to local synthesis (Björkhem and Meaney, 2004). Cholesterol in excess of requirements may either be stored as cholesteryl esters or eliminated via oxidation and/or conversion into bile acids (Björkhem, 2013). Thus, in both the cell and the intact organism, the overall cholesterol level depends on the contribution of cholesterol synthesis, absorption, elimination, and storage. This review presents a review of the current evidence of the role of epigenetic mechanisms in each of these processes.

## FUNDAMENTALS OF LIPOPROTEIN HOMEOSTASIS

Due to its insoluble nature, cholesterol cannot be solubilized in the plasma and is transported as cargo in proteolipid complexes known as lipoprotein particles. These particles consist of (i) a major structural apolipoprotein, (ii) peripheral apolipoproteins, (iii) structural lipids (phospholipids and cholesterol), and (iv) cargo lipids (highly hydrophobic lipid species such as triacylglycerols (TAG), steryl esters and fat soluble drugs, and vitamins). Lipoproteins can be divided into two broad classes – those containing Apolipoprotein B (*APOB*) and those containing Apolipoprotein A1 (*APOA1*) as core structural components. In combination with other peripheral apolipoproteins such as *APOE*, *APOCII*, and *APOCI*, these proteins define the function and metabolic fates of lipoproteins in the body (Vance and Vance, 2008).

Dietary lipids are absorbed via the small intestine and packaged into large particles known as chylomicrons (CM), which contain the intestine-specific *APOB*48, which is a truncated form of *APOB*. CM are essentially TAG delivery systems that distribute dietary lipids throughout the body for direct use and/or storage (Vance and Vance, 2008). CM remnant particles eventually return to the liver where their remaining lipid cargo, now relatively enriched in cholesterol, integrates into endogenous hepatic lipid pathways. The liver produces a second class of *APOB* containing lipoproteins, very low density lipoprotein (VLDL), which contain the full-length *APOB* and are more enriched with cholesterol than CM. In similarity with CM, VLDL participates in TAG distribution from the liver to the periphery. However, as core TAG are delivered to the tissues of the body VLDL shrink and shed peripheral apolipoproteins. During this process, the proportion of cholesterol in the particle increases progressively, ultimately leading to the production of low-density lipoprotein (LDL) particles. By binding to the LDL-receptor (LDLR) on the outer surface of expressing cells, LDL can be internalized into the cell and its cholesterol containing cargo delivered inside the cell. As an individual, LDLR is reused multiple times, LDL acts as an efficient cholesterol delivery system.

In contrast to *APOB* pathways, which are responsible for lipid transport to the periphery, *APOA1* containing lipoproteins (i.e., high-density lipoproteins, HDL) are the mediators for reverse cholesterol transport (RCT). APOA1 is mainly synthesized by the liver and intestine, although other cell such as macrophages can also produce *APOA1*. Nascent HDL particles are lipid poor and collect free cholesterol and phospholipids from peripheral tissues in an *ABCA1-*dependent process. Esterification of cholesterol by lecithin-cholesterol acyl transferase (*LCAT*) leads to an increase in core cholesteryl esters and the formation of HDL2 particles. HDL2 may be further enriched with lipids by the action of the ATP-binding cassette subfamily G member 4 (*ABCG4*). This lipid cargo may then be delivered to the liver via Scavenger Receptor B1 (*SCARB1*). As the HDL particle is not destroyed during lipid delivery to the liver, it can participate in RCT several times, enhancing the efficiency of the RCT process.

For more details on the details of lipoprotein homeostasis the reader is referred to Kingwell (Kingwell et al., 2014) and chapters 17–20 of Vance and Vance (2008).

## CHOLESTEROL SYNTHESIS

The biosynthesis of cholesterol is a highly complex process involving more than 30 different reactions and over 15 enzymes present in many different subcellular compartments (Sharpe and Brown, 2013). Conceptually, this pathway may be divided into two stages (i) condensation of isoprenoid units to yield the 30-carbon molecule squalene and (ii) cyclization of squalene to produce lanosterol, which is converted to cholesterol.

Cholesterol biosynthesis begins with the formation of the six-carbon 3-hydroxy-3-methylglutaryl-coenzyme A (HMG-CoA) from one molecule of acetyl-CoA (2C) and one of acetoacetyl-CoA. This reaction is catalyzed by the soluble enzyme 3-hydroxy-3-methylglutaryl-Co-enzyme A synthase (*HMGCS*). HMG-CoA is then converted to mevalonate by the action of the membrane bound enzyme 3-hydroxy-3-methylglutaryl-Co-enzyme A reductase (*HMGCR*) in a reaction considered to be the primary flux governing step in the cholesterol biosynthesis pathway and the target of the statin class of cholesterol lowering drugs. Recently, Gill et al. (2011) proposed that squalene epoxidase (SQLE), which catalyzes the addition of the oxygen moiety to squalene, may be a second flux governing step.

Regulation of cholesterol biosynthesis is achieved via an elegant system of feedback inhibition which senses intracellular levels of cholesterol and subsequently modulates the expression of the key proteins involved in cholesterol homeostasis (Ye and DeBose-Boyd, 2011). The master regulators of this process are the sterol response element binding proteins (*SREBF1* and *SREBF2,* also known as SREBP1 and SREBP2), which are hairpin-shaped membrane-anchored transcription factors localized to the endoplasmic reticulum (ER). Gene regulation occurs when the transcriptionally active part of the molecule (a member of the basic loop helix leucine zipper family) is released from its membrane anchor and translocates to the nucleus. This process is dependent on the levels sterols and fatty acids, although only cholesterol will be discussed here.

*SREBF* is retained in the ER due to cholesterol-dependent interactions with two proteins: SREBP cleavage activating protein (*SCAP*) and insulin sensitive gene 1 (*INSIG-1*). When cellular cholesterol levels are reduced, the amount bound to SCAP is reduced, leading to a conformational change, which allows INSIG-1 to dissociate from the SREBF–SCAP complex and is tagged for degradation by the proteosome (Ye and DeBose-Boyd, 2011). In the absence of INSIG-1, the complex then translocates to the Golgi apparatus, where is subjected to two sequential proteolytic cleavages, first by site-1 protease (*S1P*) and then by site-2 protease (*S2P*). The net result of these cleavages is that the transcriptionally active domain is released from SREBF and is free to translocate to the nucleus.

Recent evidence has highlighted a role for microRNAs (miRNAs) in cholesterol homeostasis. miRNA 33a and miR-33b are encoded by introns in the *SREBF1* and *SREBF2* genes, respectively, and are highly similar. miR-33a is co-regulated with *SREBF2*, with low cholesterol levels promoting an increase and high sterol levels promoting the opposite (Marquart et al., 2010). Very recently Yang et al. (2014) identified a potential miRNA response element for miR-185 in the promoter of *Srebf2* and demonstrated that lentiviral overexpression of this miRNA resulted in a decrease in hepatic *Hmgcr* and *Ldlr*. *In vivo*, miR-185 is regulated by *Srebp1c*, thus suggesting the presence of a complex feedback loop integrating classical mechanisms with miRNA-dependent processes. In addition, miR-520d and miR-224 both significantly suppress the mRNA expression of *Hmgcr*. Notably, these miRNAs also target various genes in cholesterol transport and lipoprotein homeostasis (cf below), indicating that they have a key role in the overall coordination of cholesterol homeostasis.

The *SREBF* proteins are known to be acetylated at conserved lysine residues by the histone acetylase cAMP responsive element binding protein 1-binding protein (*CBP*)/p300 (*EP300*, more commonly known as p300 or KAT3B; Giandomenico et al., 2003). This impedes the ubiquitination and degradation of *SREBF* and so prolongs its residence time in the nucleus, thus promoting its transcriptional activity (*ibid*). Recent evidence indicates that sirtuin 1 (*SIRT1*) can counteract this by directly deacetylating *SREBF* (Walker et al., 2010). *SREBF* may also be be phosphorylated, which creates a recognition site for the F-Box and WD Repeat Domain Containing 7, E3 Ubiquitin Protein (*FBXW7*) and allows the multiprotein SCF complex to ubiquitinate it (Sundqvist et al., 2005). Thus, the steady-state amount of transcriptionally active *SREBF* is under complex regulation at multiple levels and maintenance of active *SREBF* mediated gene transcription requires continuous cleavage of the transcriptionally active domain.

The rate of cholesterol synthesis varies considerably between different organs and tissues, and even between the cells of the same organ (Dietschy and Turley, 2002). Unsurprisingly, the expression of cholesterogenic genes varies in parallel with the rate of synthesis. A further source of variability is the age-dependency of cholesterol synthesis (Dietschy, 2009) with some cell types being very active during some periods of life and then becoming effectively quiescent afterwards (e.g., oligodendroglia). The combination of these factors leads to a unique synthesis profile in each cell. Although, the mechanism(s) underpinning this regulation has not been elucidated it is likely that epigenetic mechanisms play an important role.

Several investigators have reported that the genes involved in cholesterol biosynthesis are under epigenetic regulation, using a variety of *in vivo* and *in vitro* approaches (summarized in Table 1). Using an *in vitro* approach, Villagra et al. (2007) demonstrated that overexpression of histone deacetylase 3 (*HDAC3*) in CHO cells resulted in a 10-fold decrease of the rate of cholesterol synthesis. To rule out a confounding effect of *SREBF* mediated effects the authors used the SRD-13A cell line, which cannot cleave *SREBF* and are cholesterol auxotrophs (Rawson et al., 1999). Compared to a control cell line there was only a slight difference following HDAC overexpression, indicating the effect is primarily mediated by epigenetic mechanisms. Intriguingly, the *HDAC3* effect was observed even though *HMGCR* was slightly up- rather than downregulated indicating that the effect was not at the classical flux controlling step in cholesterol synthesis. Instead the authors presented evidence that lanosterol synthase (*LSS*) which catalyses the cyclisation of the isoprenoid 2,3-oxidosqualene to the steroid lanosterol, was markedly downregulated by HDAC3 expression or treatment with the HDACi trichostatin A (TSA). Subsequently, Knutson et al. (2008) used an *Hdac3* knockout mouse to show that loss of *Hdac3 in vivo* resulted in a de-repression of cholesterol synthesis and secretion by the liver, with several genes of the cholesterol synthesis pathway increased significantly. This lead to an age dependent increase in liver cholesterol content (fivefold increase in cholesteryl esters and a twofold increase in total cholesterol at P56) and a corresponding increase in serum total (threefold) and LDL-cholesterol (fivefold). Although there are differences in these reports, most likely due to the different experimental approaches, they are consistent in that an increase in *HDAC3* leads to a decrease in cholesterol synthesis and content, while loss of *Hdac3* leads to the opposite situation.

**Table 1:** Reported epigenetic changes in the expression of genes involved in cholesterol biosynthesis.

Experimental system	HepG2	HeLa	SH-SY5Y	Hdac3 (-/-) mouse	Mecp (-/-) mouse	Rat primary neuron	HepG2; mouse liver
Treatment	TSA	TSA	TSA	N/A	N/A	AK1	SIRT6
Reference	Chittur et al. (2008)	Villagra et al. (2007)	Nunes et al. (2012)	Knutson et al. (2008)	Buchovecky et al. (2013)	Luthi-Carter et al. (2010)	Elhanati et al. (2013)
**Gene**
*HMGCS*	↓	↑	↓	↑	–	↓	↓
*HMGCR*	↓	↑	↓	–	↓	↓	↓
*MVK*	↓	↑	↓	–	–	↓	–
*PMVK*	–	↑	–	–	–	↓	–
*MVD*	–	–	–	–	–	↓	–
*IDI1/IDI2*	–	–	–	↑	–	↓	–
*FDPS*	↓	↓	–	↑	–	↓	–
*SQS/FDFT1*	↓	↑	–	↑	–	↓	–
*SQLE*	↓	↑	–	↑	↓	↓	–
*LSS*	–	↑	–	–	–	–	–
*CYP51A1*	↓	–	–	↑	–	–	–
*SC4MOL*	↓	–	–	↑	–	↑	–
*NSDHL*	↓	–	–	–	–	–	–
*SC5D*	↓	–	–	–	–	↓	–
*DHCR7*	↓	–	–	–	–	↓	–
*DHCR24*	–	–	–	–	–	–	–

*Data is extracted from each of the above references and summarizes the direction of the changes in expression. The magnitude of the change is not indicated; – indicates that there were no data available or there was no significant change reported.*

In a more broad array based survey, Chittur et al. (2008) carried out transcriptional profiling on HepG2 hepatoma and F9 mouse embyronic carcinoma cells following treatment with TSA. They reported that numerous genes of cholesterol synthesis were downregulated by TSA and confirmed the change in expression using qPCR. *SREBF2* was also decreased, which may contribute to the changes observed in the cholesterol synthesis pathway in the cell systems studied. They did not report if cholesterol levels were altered by the treatment. Drzewinska et al. (2011) reported that 24-dehydrocholseterol reductase (*DHCR24*), which is not reported in the abovementioned studies, was induced by treatment with either TSA or 5-azacytidine (5AZA) in some, but not all, of the cell types tested. Taken together, the data available in connection with these studies indicates that epigenetic mechanisms are important for the regulation of the cholesterol synthesis pathway. Moreover, they also provide concrete evidence of cell-type sensitivity to epigenetic regulation.

As described above, members of the SIRT family are protein deaceylases that have been shown to have effects on cholesterol homeostasis. *SIRT2* has also been reported to have effects on cholesterol biosynthesis, although the data are somewhat variable. Transcriptional profiling of of primary rat neuronal cultures following treatment with small molecule *SIRT2* inhibitors (AK-1 or AGK2) for 24 h resulted in the decreased expression of many genes involved in cholesterol biosynthesis, while total sterol levels were also decreased to about two-thirds of the vehicle treated controls (Luthi-Carter et al., 2010). These investigators also reported that abundance of *SREBF* in the nucleus was reduced following SIRT inhibition, an effect that they speculated was due to a trafficking defect. However, given the sensitivity of *SREBF* to acetylation, this observation may be explained by increased degradation of nuclear *SREBF* rather than decreased trafficking. In contrast, studies using a *Sirt2* knock-out mouse failed to recapitulate these data, with no changes in the mRNA of cholesterol synthesis genes (Bobrowska et al., 2012). However, as Bobrowska et al. analyzed brain regions with mixed cell types rather than primary cultures of a single cell type, any differences (e.g., in the neuronal compartment) may have been “diluted” by more abundant cell types in which there were no change. A further confounder is the known age-dependent changes in the expression of genes involved in sterol homeostasis in neurons, with significant changes occurring in the immediate postnatal period (Ohyama et al., 2006). Extrapolation of results concerning cholesterol homeostasis from primary cultures based on embryonic cells to the adult situation should therefore be done with caution.

*SIRT6* has also been shown to be involved in cholesterol homeostasis. Elhanati et al. (2013) reported that cholesterol content in HepG2 cells was reduced (by approximately one-third) following ectopic overexpression of *SIRT6* while RNAi mediated knockdown resulted in an increase in cellular cholesterol content of the same magnitude. The mRNA expression of both *HMGCS* and *HMGCS* changed in parallel with the changes in cholesterol content. Importantly similar results were found in the livers of a mouse model overexpressing *Sirt6*. The authors provided evidence that this regulation was due to effects of *SIRT6* on *SREBF1* and *SREBF2* via a combination of direct transcriptional regulation, inhibition of the proteolytic release of the transcriptionally active form of *SREBF* and promotion of phosphorylation of SREBF by AMP activated protein kinase (*AMPK*).

The expression of miR-33a and miR-33b, causes a decrease in the expression of *SIRT6* (Elhanati et al., 2013). Effects related to mIR-dependent regulation may account for the magnitude of the equal but opposite regulation by *SIRT6* overexpression an *SIRT6* knockdown, respectively. Subsequently, Tao et al. (2013) described additional mechanistic details and highlighted a key role for the forkhead box O3 (*FOXO3*) protein in recruiting *SIRT6* to the *SREBF2* promoter, leading to histone deacetylation and reduced expression of cholesterol biosynthetic genes. Importantly, these investigators also provided independent replication of the effect of Sirt6 overexpression on cholesterol concentration *in vivo*.

Very recently Poirier presented evidence that the commonly used DNA methylation inhibitor regulator 5AZA is capable of reducing the impeding cleavage of SREBF, thus reducing the expression of HMGCR (Poirier et al., 2014). Critically these effects were independent of effects on DNA methylation. These results add an extra layer to the interpretation of the results of studies on DNA methylation and cholesterol homeostasis using 5AZA.

## CHOLESTEROL ABSORPTION

The absorption of cholesterol from the intestinal lumen is a complex and incompletely understood process (van der Wulp et al., 2013). As a very hydrophobic molecule cholesterol requires the presence of bile salts and phospholipids to form mixed micelles which permit its uptake into the enterocytes. Various proteins have been associated with inward flux of cholesterol into the enterocyte, most notably the Niemann Pick Disease Type C1 Like-1 (*NPC1L1*) protein. The heterodimer formed by the ATP binding cassette sub-family members G5 and G8 (*ABCG5* and *ABCG8,* respectively) is associated with an outward sterol flux back into the intestinal lumen. There is very limited data on the involvement of role of epigenetic mechanisms in the regulation of these key genes. Very recently, Malhotra et al. (2014) described a key role for promoter methylation in the regulation of *Npc1l1* expression in the mouse gastrointestinal tract. It is well established that *Npc1l1* expression is restricted in the gastrointestinal tract with expression in the small but not the large intestine (Davis and Altmann, 2009). The low colonic expression appears to be a result of extensive methylation of the promoter in the colon but not in the ileum or jejunum, and 5AZA treatment resulted in the derepression of *Npc1l1* expression in the colon (Malhotra et al., 2014). Furthermore, using a gene knockdown approach it was shown that both DNA methyltransferases 1 and 3B (*DNMT1* and *DNMT3B*, respectively) are involved in maintaining the hypermethylated state in the colon. Simultaneous knockdown of both was required for the depression effect which is consistent with redundancy in the regulatory process. In similarity with *NPC1L1* there are very limited data on epigenetic regulation of *ABCG5/ABCG8*, and none in the context of the intestine as far as it has been possible to determine. Studies on mouse liver carried out by Imai et al. (2009), however, indicate that the common promoter is acetylated and unmethylated, in the liver at least.

## CHOLESTEROL ELIMINATION

Cholesterol is the obligate precursor for the formation of bile acids, which are essential for the absorption of dietary lipids. Formation of bile is the major route of cholesterol elimination from the intact organism and although the pathways terminate in the liver, bile acid synthesis can be initiated in almost all cell types (Björkhem, 2013; van der Wulp et al., 2013). Two interconnected pathways which differ at the point in which the C27 carboxylic acid group is added are recognized – the classical (or neutral) pathway and the alternative (or acidic) pathway. The key structural modifications which occur are the saturation of the sterol nucleus, introduction of 2–3 hydroxyl groups in the alpha configuration and the oxidation of the side chain to yield a 24-carbon molecule. As part of these pathways a class of compounds called oxysterols (or cholesterol oxidation products) are generated, each of which contains one or more additional oxygen containing functional groups. The enzymes responsible for the formation of these oxysterols (i.e., genes responsible for oxysterol synthesis, GROS) are unevenly distributed across different cells and tissues in the body, e.g., *CYP7A1* is liver specific while *CYP46A1* is found in CNS neurons only (Meaney, 2013). In similarity with the situation for *ABCG5/ABCG8* and *NPC1L1* above, epigenetic mechanisms have been shown to have a role in the regulation of these genes. Only those genes where there is data indicating a role for epigenetic regulation will be discussed.

Cholesterol 7α-hydroxylase (*CYP7A1*) is the rate limiting step in the classical pathway of bile acid biosynthesis. Its regulation has been studied in detail and the reader is referred to Chiang (2013) for a recent review. In brief, bile acids, by acting as ligands for the nuclear hormone receptor FXR, lead to the suppression of *CYP7A1* expression via an indirect negative feedback cycle dependent on the small heterodimer partner (*SHP*) protein. From an epigenetic view point, the work of Kemper and colleagues has highlighted a key role for the farnesoid X-receptor (FXR) dependent events at the promoter of SHP – binding of ligand activated FXR to the promoter leads to the recruitment of p300 and a subsequent increase in both histone and FXR acetylation. Numerous chromatin remodeling factors – Brahma-related gene-1 (*BRG1*), Brg-1 associated factors (BAF) and activating signal cointegrator-2 containing co-activator complex (ASCOM) – are then recruited to the promoter, promoting significant chromatin remodeling, which triggers upregulation of *SHP*. *SHP* interacts with liver receptor homolog-1 (*LRH1*) at the *CYP7A1* promoter and recruits SIN3 transcription regulator family member A (*SIN3A*), HDACs1 and 2 and euchromatic histone-lysine *N*-methyltransferase 2 (*EHMT2*, also known as G9a). Ultimately a SWI/SNF related, matrix associated, actin dependent regulator of chromatin, subfamily a, member 2 (SMARCA2, also known as BRM) complex remodels the chromatin to suppresses *CYP7A1* expression (Smith et al., 2013). *SHP* has also been shown to recruit *SIRT1* to *LRH1* target genes (including *SHP* itself), leading to *SIRT1* dependent histone deacetylation and inhibition of transcription of the *CYP7A1* gene and providing a self-regulating feedback loop (Chanda et al., 2010).

*In vivo* studies indicate that *Hdac3* may also play a role in regulation of *Cyp7a1* – knockout of *Hdac3* in the mouse leads to increased histone acetylation at the *Cyp7a1* promoter and an increase in mRNA expression (Knutson et al., 2008). Treatment of mice with TSA or valproic acid (VPA) resulted in a large increase in *Cyp7a1* expression and prevented normal feedback regulation by bile acids, probably by interfering with the sophisticated mechanisms described above. In addition data has been presented that *Hdac7* was critical for the HDACi mediated increase. There is limited evidence for a role of miRNA in the regulation of *CYP7A1*. Song et al. (2010) reported that *CYP7A1* was a target for the liver enriched miR-122 as well as miR-422a and that silencing of these miRNAs induced gene expression.

Taken together this evidence indicates that epigenetic mechanisms are critical for the regulation of CYP7A1 in many species, although there is clearly more work to be done to elaborate additional details. For example, the basis for the hepatospecificity has yet to be established and the role of SIRTs requires further scrutiny.

Sterol 27-hydroxylase (CYP27A1) catalyzes the formation of both 27-hydroxycholesterol (27-OHC) and 3β-hydroxy-5-cholestenoic acid (CA) and is the initial step in the alternative pathway of bile acid biosynthesis. Although certain cell types (i.e., hepatocytes and cells of the monocytic lineage) express high levels of the enzyme it is considered relatively ubiquitous. Studies on epigenetic regulation of CYP27A1 are limited and are descriptive only – 5AZA was shown to have no effect in macrophages (Escher et al., 2005) while TSA treatment of HepG2 cells resulted in a six-fold decrease of *CYP27A1* mRNA at 24 h followed by a return to basal levels by 48 h (Chittur et al., 2008). In contrast, treatment of SH-SY5Y neuroblastoma cells with TSA caused a five-fold increase in expression (Shafaati et al., 2009). While it is difficult to reconcile these findings in the absence of additional mechanistic details, it is highly likely that cell specific factors are involved.

Cholesterol 24S-hydroxylase (*CYP46A1*) catalyzes the formation of 24S-hydroxycholesterol (24S-OHC), a key process for maintenance of overall cholesterol homeostasis within the brain of vertebrates. It is well established that *CYP46A1* is regulated by histone acetylation status – treatment with TSA *in vitro* results in a large increase in mRNA expression (Ballas et al., 2005; Milagre et al., 2008; Shafaati et al., 2009). Under *in vivo* conditions, treatment of mice with TSA leads to a modestly increased level of *Cyp46a1* in the liver but not the brain (Shafaati et al., 2009). Rodrigues et al. have carried out an elegant series of experiments designed to uncover the details of the mechanism underpinning this effect. Specificity proteins (SP) 1, 3, 4 appear to be critical for the effect of HDACi – loss of *SP3* from the promoter triggers a decrease in the occupancy of HDACs 1 and 2, with subsequent recruitment of *CBP* and p300 (Milagre et al., 2012). In a subsequent study these authors identified a potential role for *MEK*-*ERK* signaling in the regulatory process and provided evidence that pERK is present at the *CYP46A1* promoter where it phosphorylates *SP3*, thus triggering recruitment of corepressors (Nunes et al., 2012). Given the highly restricted expression of CYP46A1 – in healthy brain it is only present in neurons – this mechanism may represent a supression mechanism important for cell-specificity of *CYP46A1*. In addition, the RE1-silencing transcription factor (*REST*, also known as NRSF) may play a role in the suppression of CYP46A1 in non-neuronal cells (Meaney, 2013). Although the *CYP46A1* promoter is GC rich and contains several predicted CpG islands it does not appear that these are methylated. However, treatment with *DNMT* inhibitors lead to an increase in the expression of *CYP46A1*, by a mechanism involving an *SP3* dependent loss of *SIN3A* and HDAC(s) (Milagre et al., 2010). In the *Mecp2* null mouse, expression of *CYP46A1* was markedly decreased with a concomitant reduction in the rate of cholesterol synthesis rate (Buchovecky et al., 2013). These data indicate that epigenetic mechanisms are critical for the regulation of brain cholesterol balance and may indeed represent critical coordination point between brain cholesterol synthesis and eliminations.

Cholesterol 25-hydroxylase (*CH25H*) catalyses the synthesis of 25-hydroxycholesterol (25-OHC), a molecule which has very recently been described to have potent anti-viral and paracrine immunomodulatory effects (Bauman et al., 2009; McDonald and Russell, 2010; Liu et al., 2013). It has been shown that the signal transducers and activators of transcription 1 (*STAT1*) pathway can regulate *Ch25h* in mice (Blanc et al., 2013). As HDAC activity is required for *STAT1* signaling, epigenetic mechanisms likely contribute to this process. Gold et al. (2012) demonstrated that deletion of the activating transcription factor-3 (*Atf3*) gene lead to increased *Ch25h* promoter acetylation in bone marrow derived macrophages, with the consequence of increased mRNA and 25-OHC.

In contrast to the other genes responsible for the formation of oxysterols, CYP3A4 encodes an enzyme with a primary responsibility for the metabolism of pharmaceuticals and xenobiotics. In addition, it has been reported that CYP3A4 can also produce both 4β- and 25-hydroxycholesterol (Honda et al., 2011; Diczfalusy, 2013). CYP3A4 is hepatospecific and its expression can be induced significantly, with the pregnane X-receptor acting as a key mediator of regulation. Currently available data indicates that DNA methylation plays a role in the regulation of *CYP3A4* – treatment with 5^′^-aza-deoxycytidine increased the expression of *CYP3A4* in a variety of cell lines, with induction in some cell systems by a factor of more than 100-fold (Dannenberg and Edenberg, 2006; Habano et al., 2011). Due to the sensitivity of *CYP3A4* to *PXR*, these effects are likely due to differential methylation at the promoter of *PXR* rather than a direct effect on *CYP3A4*. In studies on the mouse ortholog *Cyp3a11*, Li et al. (2009) demonstrated that increased expression of *Cyp3a11* was associated with increased histone H3 lysine-9 dimethylation. In the brain, which does not express *Cyp3a11*, there was extensive histone-H3 lysine-9 dimethylation and lysine-27 trimethylation at the *Cyp3a* locus (Mikkelsen et al., 2007). At a mechanistic level, it has been shown that ligand binding to *PXR* triggers recruitment of protein arginine N-methyltransferase-1 (*PRMT1*) to the *CYP3A4* promoter which triggers increased histone acetylation and methylation (Xie et al., 2009).

Oxysterol 7α-hydroxylase (*CYP7B1*) is responsible for the 7α-hydroxylation of intermediates in bile acid synthesis such as 27-OHC and 25-OHC but not 24S-OHC (*cf* below). In similarity with 27-OHC, *CYP7B1* is considered to be an ubiquitous enzyme although its expression is greater in males than in females (Leuenberger et al., 2009; Stiles et al., 2009). It is known that the *CYP7B1* promoter is methylated and treatment of the prostate cancer cell line LNCaP with *DNMT* inhibitors leads to a reduction in the mRNA levels (Olsson et al., 2007). The results following HDACi treatment are more variable and appear to be dependent on the cell type – HDACi treatment leads to a reduction in *SH-SY5Y* cells but an induction in *HepG2* cells (Chittur et al., 2008; Shafaati et al., 2009). This latter induction was shown to be dependent on *HDAC1* and *HDAC3* dependent processes. Methylation has a key role on the sexual dimorphic expression of *CYP7B1* – sumoylation of the nuclear hormone receptor peroxisome-proliferator activated receptor triggers enhanced interaction with GA-binding protein (*GABP*) and the recruitment of various *HDAC* and *DNMT.* The net effect is the methylation of an important *SP1* binding site in the *CYP7B1* promoter, preventing its binding to the promoter and so the expression is reduced (Leuenberger et al., 2009).

24S-hydroxycholesterol 7α-hydroxylase (*CYP39A1*) is responsible for the 7α-hydroxylation of the 24S-OHC. It has limited expression throughout the body, with liver, eye and brain expression reported (Li-Hawkins et al., 2000; Steckelbroeck et al., 2002; Ikeda et al., 2003; Shafaati et al., 2009). In similarity with *CYP7B1*, *CYP39A1* is expressed in a sexually dimorphic manner, with higher levels in females compared to males (Li-Hawkins et al., 2000). Data on the epigenetic regulation of *CYP39A1* are very limited – HDACi treatment in mice results in an increase in *Cyp39a1* in the brain while at the same time decreasing hepatic expression (Shafaati et al., 2009). Huang et al. (2009) have described that *CYP39A1* may be hypermethylated in ovarian cancer, but did not report effects on mRNA levels. Using a hypomorphic *DNMT1* mouse line, Kutay et al. (2012) recently reported that the expression change in the liver of *Cyp39a1* in response to alcohol challenge was blunted by more than half in the *DNMT1* deficient lineage.

## LIPOPROTEINS

To date, there have been few reports on the epigenetic regulation of plasma lipoproteins or the proteins involved in their turnover and there do not appear to be any regulation of the structural apolipoproteins APOA1 and APOB. However, there is some recent evidence that *APOE*, which is present on both *APOB* and *APOA1* containing lipoproteins in the circulation, is epigenetically regulated. In an early study on hyperhomocystenemia Yi-Deng et al. (2007) reported a decrease in *Apoe* mRNA and protein following homocysteine treatment, a decrease which was reversed with folate treatment. These changes were matched by an concomitant increase promoter methylation, linking the change to differential methylation of the DNA. Very recently, Yu et al. (2013) reported that exon 4 of the *APOE* gene, which contains the important e2/e3/e4 polymorphic region, contained a CpG island. This region was highly methylated in numerous brain regions and also in peripheral blood lymphocytes and the degree of methylation appeared to be sensitive to *APOE* allele present in exon 4. Moreover, there were functional consequences – the differentially methylated alleles had independent enhancer/silencer effects (Yu et al., 2013). Thus, although the direct data is limited, it appears that methylation at least plays a key role in the expression of *APOE*.

Apolipoprotein J (*CLU*, initially described as clusterin) is found in *APOA1* (i.e., HDL-type) containing-lipoproteins. Salminen’s group has studied the epigenetic regulation of *CLU* in various cellular systems. Treatment with different HDACi increased the mRNA expression of *CLU* in normal human astrocytes, C6 glioma cells and both SK-N-AS and HN10 neuroblastoma cells (Nuutinen et al., 2005). The expression in SK-N-AS cells was further enhanced by a combination of HDACi and *DNMT* inhibition. Intracellular and secreted CLU was also significantly increased by HDACi treatment (Suuronen et al., 2007). Taken together these results indicate that epigenetic mechanisms play an important role in the regulation of CLU.

In HepG2 cells, HDACi treatment appears to reduce the expression of the LDL-receptor (Chittur et al., 2008; Nunes et al., 2012). In addition, the proprotein convertase subtilisin/kexin type 9 protein (*PCSK9*), which controls the degradation of the LDL-receptor and is a critical regulator of the amount of *LDLR* expressed on the cell surface, is epigenetically regulated. *SIRT6* is recruited to the promoter by *FOXO3* and promotes the deacetylation of histone H3 proteins at lysines 9 and 56 (Tao et al., 2013). This has the effect of reducing PCSK9 expression, preserving LDL-R exposure and function and thus lowering plasma low density lipoprotein cholesterol levels. In the Sirt6 knockout mouse, *Pcsk9* levels are elevated with a concomitant reduction in the expression of the Ldlr and an increases the level of cholesterol in the blood (*ibid*).

Intriguingly, some very recent data indicates that lipoproteins may trigger epigenetic changes in genes involved in disease. Kumar et al. (2013) demonstrated that *DNMT1* expression and activity were induced in endothelial cells following treatment with LDL cells. Binding of *MECP2* was also enhanced, with a net result of a decrease in endothelial Kruppel-like factor 2 (*KLF2*) expression. The overall effect was an enhanced procoagulant status of the endothelium a change which may have functional significance (*ibid*).

Accumulating evidence indicates that microRNAs (miRNA) are involved in the regulation of lipoprotein homeostasis, adding an additional level of complexity and control to the process. Studies by Esau et al. (2006) identified that miR-122 was highly enriched in the liver and was able to regulate plasma cholesterol in the mouse and in non-human primates. Ablation of miR-122 using antisense oligonucleotides resulted in a decrease in total cholesterol by 25–30%, with the effect in non-human primates due to a reduction in LDL cholesterol. However, the mechanism for these changes was not obvious, despite the use of genome wide expression profiling approaches. miR-370 has also been shown to indirectly influence plasma cholesterol levels, via modulation of miR-122 levels (Iliopoulos et al., 2010). On the other hand, several miRNAs – including miR-33a/b, miR-128, and miR-144 – have been reported to modulate the expression of components of the pathway for HDL-dependent cholesterol eﬄux. This regulatory mechanism appears to be of functional importance, as silencing of these miRNAs *in vivo* leads to an increase in plasma HDL (Rayner et al., 2011a; Adlakha et al., 2013; Ramírez et al., 2013). This may represent a regulatory mechanism which converges on the flux-governing steps of sterol eﬄux.

Salerno et al. recently described two miRNAs which appear to have a role in regulation of the *LDLR* – miR-520d and miR-224 both target genes involved in determining how much *LDLR* is expressed on the cell surface, namely *IDOL* and *PCSK9* (Salerno et al., 2013). *In vitro* overexpression of miR-520d decreased the expression of both genes by 30–50%, while similar experiments with miR-224 resulted in a decrease in the order of 75%. The net effect of these decreases was an increase in the cell-surface expression of the *LDLR* and greater LDL binding.

## CHOLESTEROL STORAGE

Excess cholesterol in the cell can be converted to choelsteryl esters by sterol *O*-acyl transferases 1 and 2 (*SOAT1* and *SOAT2*, respectively, also known as ACAT1 and ACAT2). This provides a mechanism whereby cells can store cholesterol and remove it from the metabolically active pool present in the cell. Data on the regulation of *SOAT1* and *SOAT2* are very limited and is found only in studies examining other aspects of lipid metabolism. Tessema and Belinsky (2008) identified that the *SOAT2* promoter was methylated to varying degrees among a panel of lung cancer adenocarcinoma cells. Liang et al. (2013) reported a modest decrease in *SOAT1* promoter methylation following homocysteine treatment, which was contemporaneous with an increase in *SOAT1* mRNA and cholesteryl ester concentration. Finally, Devlin et al. (2010) reported that hepatic expression of *Soat2* in hyperhomocystenemic mice was reduced in the context of elevated DNA methylation. Thus, although the available data is limited, there are indications that sterol esterification is likely to be under epigenetic regulation.

## POTENTIAL OF EPIGENETIC THERAPIES IN DYSLIPIDEMIAS

There is relatively limited direct data on the potential for HDACi and related epigenetic therapies to influence lipid levels and the data that is available is typically in connection with consumption of juices and other supplements. In an early study, Murashima et al. (2004) examined the effects of 1-week intake of broccoli sprouts – which rich in the phytochemical HDACi sulforaphane – on various biochemical parameters in man and reported positive effects on HDL-cholesterol. Subsequent studies of hypercholesterolemic men treated with kale juice (also rich in sulforaphane) in demonstrated similar beneficial increases in HDL-cholesterol and a significant and clinically relevant decrease in the atherogenic index (Kim et al., 2008). In a randomized double-blind placebo-controlled study on type 2 diabetic patients, using broccoli sprout powder (BSP) Bahadoran et al. (2012) reported a significantly higher HDL-cholesterol in the group receiving the highest dose of BSP and a reduction in the atherogenic index similar to that described by Kim et al. (2008). Thus, although the data is both limited and confounded by the well known anti-oxidant effects of sulforaphane, it does appear that targeted dietary epigenetic therapies may be viable options for treatment of dyslipidemia.

The key role for miR-33 in regulation of cholesterol eﬄux has prompted several investigators to explore the possibility that antagonism of miR-33 may be a viable HDL-raising strategy. Initial studies from Moore’s group demonstrated that treatment of atherprone mice with anti-miR-33 lead to an increase in plasma HDL levels with no obvious hepatotoxicity and moderate regression of atherosclerosis (Rayner et al., 2011b). Subsequent studies where non-human primates were treated for a total of 12 weeks demonstrated an elevation in *Abca1* expression and increase in HDL levels (to 1.5-fold of the mismatch control), again with no apparent hepatotoxicity (Rayner et al., 2011a). Similar changes were also reported by Rottiers et al. (2013). Inhibition of the activity of miR-33 thus appears to be a potential mechanism to enhance reverse cholesterol transport *in vivo*. However, very recently Goedeke et al. (2014) reported that long-term inhibition of miR-33 in the setting of a high-fat diet may lead to hepatic steatosis. Although these studies were carried out in mice, they do indicate that anti-miR-33 therapies may have adverse effects when used for long term treatment.

Due to its hydrophobic nature, the movement of cholesterol within the cells relies on a wide variety of membrane bound and soluble proteins. The Niemann Pick Disease Type C proteins 1 and 2 (NPC1 and NPC2, respectively) work in a coordinated manner to ferry cholesterol from the endolysosomal system to the endoplasmic reticulum (Yu et al., 2014). There is compelling evidence that both of these genes are regulated by histone acetylation status and treatment of cells with a variety of different HDACi leads to a significant increase in the expression of these genes (Gévry et al., 2003; Kim et al., 2007; Pipalia et al., 2011; Helquist et al., 2013; Maceyka et al., 2013). The increase in the expression of the mutated NPC proteins, which have some by not sufficient activity, resulted in a quantitative increase in activity which was sufficient to correct the cellular defect (Munkacsi et al., 2011; Pipalia et al., 2011; Yang et al., 2014). Due the success of the HDACi treatment in pre-clinical models, HDACi therapy is currently being trialed in a small group of NPC patients. This underscores the potential for therapies based on epigenetic approaches to address deficits in cholesterol homeostasis which are currently intractable by other approaches.

## CONCLUDING REMARKS

Epigenetic regulation of cholesterol and sterol homeostatic genes is a burgeoning field which is already showing great potential to explain the behavior of genes involved in sterol balance. There are numerous questions outstanding in the field, but there are excellent investigators working intensively to address them. The goal will be to translate basic research findings into transcriptional therapies for non-cancer human diseases, in a similar manner to NPC disease as outline above. The availability of pharmaceutical compounds developed for other diseases (e.g., vorinostat) as well as the ever increasing amount of dietary epigenetic modulators provides a good foundation for translation into the clinical setting as possible therapies for cardiovascular and neurodegenerative diseases. The efforts of dedicated investigators who continue to shed light on the importance of epigenetic mechanisms will contribute to this development in the coming years.

## Conflict of Interest Statement

The author declares that the research was conducted in the absence of any commercial or financial relationships that could be construed as a potential conflict of interest.

## Footnotes

1 It should be noted that cholesterol itself is not a direct precursor for vitamin D; rather the sterol 7-dehydrocholesterol, itself an intermediate in cholesterol biosynthesis, fulfils this role.
